# Acute Chest Syndrome

**DOI:** 10.21980/J80S8J

**Published:** 2023-01-31

**Authors:** Patrick Meloy, Daniel R Rutz, Amit Bhambri

**Affiliations:** * Emory University School of Medicine, Department of Emergency Medicine, Atlanta, GA; ^ University of Wisconsin-Madison School of Medicine and Public Health, Department of Emergency Medicine, Madison, WI; † Swedish Hospital, Part of Northshore, Department of Emergency Medicine, Chicago, IL

## Abstract

**Audience:**

Emergency medicine residents and medical students on emergency medicine rotations

**Introduction:**

Acute chest syndrome is a life-threatening, potentially catastrophic complication of sickle cell disease.^1^,^2^ It occurs in approximately 50% of patients with sickle cell disease, with up to 13% all-cause mortality.^1^ Most common in children aged 2–4, up to 80% of patients with a prior diagnosis of acute chest syndrome will have recurrence of this syndrome.^4^ Diagnostic criteria include a new infiltrate on pulmonary imaging combined with any of the following: fever > 38.5°C (101.3°F), cough, wheezing, hypoxemia (PaO2 < 60 mm Hg), tachypnea, or chest pain.^4^,^5^ The pathophysiology of acute chest syndrome involves vaso-occlusion in pulmonary vessels resulting in hypoxia, release of inflammatory mediators, acidosis, and infarction of lung tissue. The most common precipitants are infections (viral or bacterial), rib infarction, and fat emboli.^1^,^2^,^4^ Patients commonly present with fever, dyspnea, cough, chills, chest pain, or hemoptysis. Diagnosis is made through physical exam, blood work, and chest imaging.^1^,^2^ Chest radiograph is considered the gold standard for imaging modality.^3^ Management of acute chest syndrome includes hydration with IV crystalloid solutions, antibiotics, judicious analgesia, oxygen, and, in severe cases, transfusion.^6^ Emergency medicine practitioners should keep acute chest syndrome as a cannot miss, high consequence differential diagnosis for all patients with sickle cell disease presenting to the Emergency Department.

**Educational Objectives:**

At the end of this oral board session, examinees will: 1) demonstrate the ability to obtain a complete medical history; 2) demonstrate the ability to perform a detailed physical examination in a patient with respiratory distress; 3) identify a patient with respiratory distress and hypoxia and manage appropriately (administer oxygen, place patient on monitor); 4) investigate the broad differential diagnoses which include acute chest syndrome, pneumonia, acute coronary syndrome, acute congestive heart failure, acute aortic dissection and acute pulmonary embolism; 5) list the appropriate laboratory and imaging studies to differentiate acute chest syndrome from other diagnoses (complete blood count, comprehensive metabolic panel, brain natriuretic peptide (BNP), lactic acid, procalcitonin, EKG, troponin level, d-dimer, chest radiograph); 6) identify a patient with acute chest syndrome and manage appropriately (administer intravenous pain medications, administer antibiotics after obtaining blood cultures, emergent consultation with hematology) and 7) provide appropriate disposition to the intensive care unit after consultation with hematology.

**Educational Methods:**

This case is used as a method to assess learners’ ability to rapidly assess a patient in respiratory distress. The learner needs to address a limited differential diagnosis list while simultaneously stabilizing and treating the patient. The “patient” becomes an active participant in the case, with repeated requests for pain medication, and appropriate analgesic administration is required as a critical action. For faculty, this case is used to assist with periodic assessment of resident performance while in the emergency department (ED).

We use oral board testing as one additional tool to assess residents’ critical thinking, while still applying the pressure that is needed to pass the oral certification examination. Large groups of residents can be assessed in short periods of time without needing to “wait” for this particular patient presentation to be seen in the ED.

In this case, learners were assessed using a free online evaluation tool, Google forms. Multiple questions were written for each critical action, and the Google form served as the online evaluation and repository of this information. The critical actions of the case were then tied to Emergency Medicine Milestones, and the results were compiled for use during resident clinical competency evaluations. Residents were provided with immediate feedback of their performance and were also given their electronic evaluations when requested.

**Research Methods:**

To assess the strengths and weaknesses of the case, learners and instructors were given the opportunity to provide electronic feedback after the case was completed. Subsequent modifications were made based on the feedback provided. Additionally, learners answered written multiple-choice questions after the case to assess for retention of the material.

**Results:**

Senior and junior residents alike enjoyed the process of an oral board simulation as an alternative to a more formal lecture. Seniors also stated that they felt more confident with their ability to pass the oral certification examination after having gone through oral board testing while in residency. Overall, the case was rated relatively highly, with residents scoring the case as 4.3 ± 0.186, 95% confidence interval (1–5 Likert scale, 5 being excellent, n=53) after their assessment was completed.

**Discussion:**

Students and residents who participated in the oral board exam formatting found this to be preferable to a traditional lecture and enjoyed the learning environment. Faculty also found this type of participation to be more engaging and were pleased with the ability to perform high-stress assessments with low stakes. The content contained in the case is relevant to all emergency medicine trainees, and this formatting forces the learner to be an active participant in the learning session. The case is a good model for the high-stakes testing of the oral certification exam and is an effective way to test a resident’s ability to rapidly assess and manage a life-threatening condition in the ED.

**Topics:**

Sickle cell anemia, vaso-occlusive pain crisis, acute chest syndrome, hypoxia, pneumonia, sepsis.

## USER GUIDE

**Table t1-jetem-8-1-o1:** 

List of Resources:	
Abstract	1
User Guide	4
For Examiner Only	6
Oral Boards Assessment	11
Stimulus	14
Debriefing and Evaluation Pearls	23

[Table t1-jetem-8-1-o1] **Learner Audience:**

Medical students, interns, junior residents, senior residents

**Time Required for Implementation:**

Case: 15 minutes as a single case

Debriefing: 10 minutes

**Learners per instructor:**

1

**Topics:**

Sickle cell anemia, vaso-occlusive pain crisis, acute chest syndrome, hypoxia, pneumonia, sepsis.

**Objectives:**

By the end of this oral boards case, examinees will be able to:

1. Demonstrate the ability to obtain a complete medical history
2. Demonstrate the ability to perform a detailed physical examination in a patient with respiratory distress
3. Identify a patient with respiratory distress and hypoxia and manage appropriately (administer oxygen, place patient on monitor)
4. Investigate the broad differential diagnoses which include acute chest syndrome, pneumonia, acute coronary syndrome, acute congestive heart failure, acute aortic dissection and acute pulmonary embolism
5. List the appropriate laboratory and imaging studies to differentiate acute chest syndrome from other diagnoses (complete blood count, comprehensive metabolic panel, BNP, lactic acid, procalcitonin, EKG, troponin level, chest radiograph)
6. Identify a patient with acute chest syndrome and manage appropriately (administer intravenous pain medications, administer antibiotics after obtaining blood cultures, emergent consultation with hematology)
7. Provide appropriate disposition to the intensive care unit after consultation with hematology
8. medicine, and we feel this case is an important addition to any oral board session.

### Linked objectives and methods

The learner in this case must be able to synthesize available history, physical examination and imaging findings (Objectives 1, 2 and 5) in order to develop a broad differential of a patient who is experiencing acute chest syndrome (Objective 4). If improperly interpreting pulse oximetry and imaging, diagnosis may be missed if the learner does not identify the patient with acute chest syndrome (Objective 3, 4 and 5). The oral board formatting allows the learner to interpret the chest radiograph in real-time in order to identify a pulmonary emergency (Objective 5). The learner must be able to diagnose acute chest syndrome and provide timely and appropriate treatment and disposition to prevent an adverse outcome (Objective 6 and 7). Debriefing of the case immediately afterward ensures assimilation of the sources of data in order to obtain the correct diagnosis and appropriate management of the case.

### Recommended pre-reading for instructor

Review references prior to case administration; no pre-reading should be required for instructors.

### Results and tips for successful implementation

This case was best used as an oral board examination. The learner should be directly observed by the examiner, either inperson or via video conference, and additional learners can be present to observe. Learners were tested during emergency medicine conferences, a mock oral board examination, and oral board practice sessions. Assessment forms were created using Google forms (http://docs.google.com/forms) and these were tied to the Emergency Medicine Milestones (https://www.acgme.org/Portals/0/PDFs/Milestones/EmergencyMedicineMilestones.pdf?ver=2015-11-06-120531-877). Using this method, the oral board formatting could assess a resident’s clinical ability to practice in a non-threatening environment, but also evaluate their progress along the ACGME’s milestones.

This case presented a challenge for medical students and interns, and also is able to test efficiency and higher-level reasoning of the senior residents. In general, learners were able to successfully obtain the diagnosis; however, there were some issues with more junior learners adding additional testing and workup when faced with hypoxia in a sickle cell patient.

After the mock oral board session was completed, residents were afforded the opportunity to rate the case individually, and this case scored 4.3, ± 0.186 (95% confidence interval), using a 1–5 Likert scale, 5 being excellent (n=53). This is consistent with previous oral board submissions. Residents were also asked to leave comments on the case using open-ended questions. Learners felt that the oral board session presented a “somewhat quick diagnosis,” in a “common presentation, but sicker than usual” patient. This concept is highly testable in emergency

## Figures and Tables

**Table:** 

**Vitals:** HR 111	BP 99/65	RR 26	Temp 37.9°C	O_2_Sat 92% on room air

**Table:** 

	Milestone	Did not achieve level 1	Level 1	Level 2	Level 3
1	Emergency Stabilization (PC1)	□ Did not achieve Level 1	□ Recognizes abnormal vital signs	□ Recognizes an unstable patient, requiring intervention Performs primary assessment Discerns data to formulate a diagnostic impression/plan	□ Manages and prioritizes critical actions in a critically ill patient Reassesses after implementing a stabilizing intervention
2	Performance of focused history and physical (PC2)	□ Did not achieve Level 1	□ Performs a reliable, comprehensive history and physical exam	□ Performs and communicates a focused history and physical exam based on chief complaint and urgent issues	□ Prioritizes essential components of history and physical exam given dynamic circumstances
3	Diagnostic studies (PC3)	□ Did not achieve Level 1	□ Determines the necessity of diagnostic studies	□ Orders appropriate diagnostic studies Performs appropriate bedside diagnostic studies/procedures	□ Prioritizes essential testing Interprets results of diagnostic studies Considers risks, benefits, contraindications, and alternatives to a diagnostic study or procedure
4	Diagnosis (PC4)	□ Did not achieve Level 1	□ Considers a list of potential diagnoses	□ Considers an appropriate list of potential diagnosis May or may not make correct diagnosis	□ Makes the appropriate diagnosis Considers other potential diagnoses, avoiding premature closure
5	Pharmacotherapy (PC5)	□ Did not achieve Level 1	□ Asks patient for drug allergies	□ Selects an appropriate medication for therapeutic intervention, considering potential adverse effects	□ Selects the most appropriate medication(s) and understands mechanism of action, effect, and potential side effects Considers and recognizes drug-drug interactions
6	Observation and reassessment (PC6)	□ Did not achieve Level 1	□ Reevaluates patient at least one time during the case	□ Reevaluates patient after most therapeutic interventions	□ Consistently evaluates the effectiveness of therapies at appropriate intervals
7	Disposition (PC7)	□ Did not achieve Level 1	□ Appropriately selects whether to admit or discharge the patient	□ Appropriately selects whether to admit or discharge Involves the expertise of some of the appropriate specialists	□ Educates the patient appropriately about their disposition Assigns patient to an appropriate level of care (ICU/Tele/Floor) Involves expertise of all appropriate specialists
22	Patient centered communication (ICS1)	□ Did not achieve level 1	□ qEstablishes rapport and demonstrates empathy to patient (and family) Listens effectively	□ Elicits patient’s reason for seeking health care	□ Manages patient expectations in a manner that minimizes potential for stress, conflict, and misunderstanding.
23	Team management (ICS2)	□ Did not achieve level 1	□ Recognizes other members of the patient care team during case (nurse, techs)	□ Communicates pertinent information to other healthcare colleagues	□ Communicates a clear, succinct, and appropriate handoff with specialists and other colleagues Communicates effectively with ancillary staff

**Table:** 

#1	Patient Information Form
#2	Arterial Blood Gas
#3	CBC
#4	BMP
#5	Cardiac Labs
#6	Coagulation Panel
#7	Chest Radiograph (CXR)
#8	Electrocardiogram (EKG)

**Table:** 

Patient’s Name:	Marcus Jones
Age:	19 years old
Gender:	Male
Chief Complaint:	Body Aches
Person Providing History:	Patient

**Table:** 

Temp:	37.9°C
BP:	99/65
P:	111
RR:	26
Pulse Ox:	92% on room air
Weight:	72 kg

**Table:** 

pH	7.35
pCO2	32 mmHg
pO2	70 mmHg
HCO3	22 mmol/L
O2 sat	75%

**Table:** 

White blood cell count (WBC)	19.0 x1000/mm3
Hemoglobin (Hgb) 7.6 g/dL	
Hematocrit (Hct)	23.3%
Platelets	98 x1000/mm3

**Table:** 

Neutrophils	73%
Lymphocytes	12%
Monocytes	4%
Eosinophils	4%
Bands	7%
Reticulocyte Count	10.6% (ref: 0.5 – 2.5%)

**Table:** 

Sodium	138 mEq/L
Potassium	4.8 mEq/L
Chloride	108 mEq/L
Bicarbonate	19 mEq/L
Blood Urea Nitrogen (BUN)	26 mg/dL
Creatinine (Cr)	0.9 mg/dL
Glucose	99 mg/dL

**Table:** 

Troponin	0.04 mcg/L (ref: <0.05 mcg/L)
D-dimer	644 ng/mL (ref: <500 ng/mL)

**Table:** 

Prothrombin Time (PT)	29.1 seconds
Partial Thromboplastin Time (PTT)	15.1 seconds
International Normalized Ratio (INR)	1.1

**Figure f1-jetem-8-1-o1:**
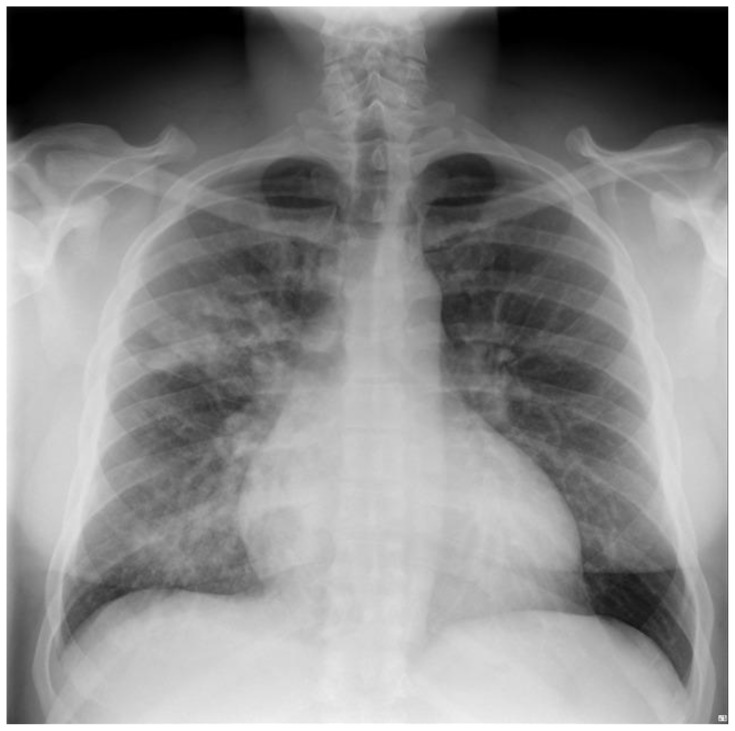
Image Source: Knipe, H. Bronchopneumonia. Case study, Radiopaedia.org. (accessed on 27 May 2022) https://doi.org/10.53347/rID-49869. CC-BY-NC-SA 3.0

**Figure f2-jetem-8-1-o1:**
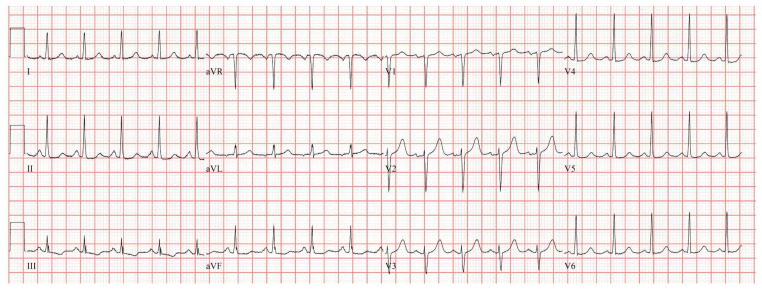
Image Source: de Jong, J. Sinus Tachycardia. ecgpedia.org. (accessed on 27 May 2022) https://en.ecgpedia.org/wiki/File:Sinustachycardia.jpg. CC-BY-NC-SA 3.0

## FOR EXAMINER ONLY

### Oral Case Summary

**Diagnosis: Acute Chest Syndrome**

**Case Summary:** This is a 19-year-old male patient with a previous history of sickle cell anemia, who is presenting to the ED with complaints of body aches. He states that he is usually able to tolerate the pain at home with his oral medications, but it became worse and now unbearable over the last three days. He usually takes oral oxycodone for his symptoms, and also has morphine for break-through pain, but he ran out of this medication. He felt some increasing shortness of breath during the day today. He is requesting Dilaudid, and states he usually needs 2mg IV push for pain relief. He also describes pleuritic bilateral chest pain. He is ultimately found to have an acute vaso-occlusive crisis with associated acute chest syndrome. He should be admitted to the MICU for ongoing care given his oxygen requirements. Hematology should be consulted, though they will not offer additional treatment plans.

**Order of Case:** This is a case of a 19-year-old male patient who is presenting to the ED with body aches. Upon arrival, he is found to be hypoxic, and should be triaged to the resuscitation room for his respiratory distress. He should be given immediate oxygen by either nonrebreather (though any type of oxygen administration will satisfy this critical action). When oxygen is given, repeat vital signs will indicate improvement in his oxygen saturation. If oxygen is not provided, repeat vital signs should indicate worsening hypoxia, with oxygen saturation 87% on repeat evaluation. The patient should be made to become more tachypneic. It is up to the examiner to determine if the patient requires non-invasive positive pressure oxygen administration (BiPAP or CPAP), though this will not be a critical action.

The patient also is noted to be in distress secondary to his painful crisis. He should be medicated with opiate analgesia; otherwise, he should continue to complain of body pain and worsening chest pain until this is addressed. It should be difficult to assess the patient for additional history or concerns until his pain is addressed, and this is a critical action of the case.

Ultimately, the patient is diagnosed with acute chest syndrome. He should be started on broad spectrum antibiotics, and cultures should be obtained while in the ED. Hematology should be consulted, although they will agree with the standard of care treatment and not provide additional information. Residents may elect to assess for pulmonary embolism (PE), which is not unreasonable, though the diagnosis is clinched with his chest radiograph. The patient should be admitted to the MICU given his oxygen requirements, sepsis and respiratory distress.

**Disposition:** Admit to intensive care unit

**Critical Actions:**

1. Patient requires oxygen administration and should concurrently be placed in a resuscitation room.
2. Patient requires IV access with appropriate analgesic administration. This includes an IV opiate medication and is not satisfied with oral medications or IV NSAIDs.
3. Patient should be started on broad spectrum antibiotics after viewing his chest radiograph, and blood cultures should be obtained.
4. Consult placed to hematology.
5. Admit to the medical intensive care unit.

### Historical Information

**Chief Complaint:** Body aches

**History of present illness:** Patient states he has been having worsening body aches for the last three to four days. He states that he is usually able to control symptoms with his home oxycodone and morphine. He states his pain is starting in his legs and radiating into his upper body. He also complains of chest pain and shortness of breath. His chest pain is bilateral and pleuritic. He interrupts the history and physical exam several times in order to ask for 2mg of IV Dilaudid. He has had a dry cough for one day. He denies sore throat, loss of taste or smell. He is not aware of any sick contacts. He is up-to-date on his vaccinations. He has not had abdominal pain, nausea, vomiting or diarrhea. He denies other symptoms.

**Past Medical history:** Sickle cell anemia

**Past Surgical history:** None

**Patient’s Medications:** Morphine and oxycodone

**Allergies:** No known drug allergies

**Social history:**

- Alcohol: Occasional
- Tobacco: Cigars, 2–3 per week for 2 years
- Drug use: Denies

**Family history:**

- Sickle cell anemia
- Hypertension
- Coronary artery disease

### Physical Exam Information

**Weight:** 72.0 kg

**General appearance:** Well-nourished, well-developed, appears in moderate distress

**Primary survey:**

- **Airway:** Intact
- **Breathing:** Hypoxic, rales at the bilateral bases, breath sounds equal bilaterally, tachypneic
- **Circulation:** Tachycardic, borderline hypotensive, intact peripheral pulses

**Physical examination:**

- **General appearance:** Well-nourished, well-developed, in moderate distress; does not wish to continue the examination until he receives pain medication
- Head, eyes, ears, nose and throat (HEENT):
  ○ **Head:** Normal
  ○ **Eyes:** Trace, bilateral scleral icterus
  ○ **Ears:** Normal
  ○ **Nose:** Normal
  ○ **Oropharynx/Throat:** Mucous membranes dry, otherwise normal
- **Neck:** Normal
- **Chest:** Rales at the bases bilaterally, tachypneic, labored breathing, sternal retractions, breath sounds diminished on the right compared to the left, no wheezes
- **Cardiovascular:** Tachycardic, +s1/s2, no murmurs or rubs, normal and symmetric peripheral pulses, brisk capillary refill in all digits, no jugular vein distention (JVD)
- Abdominal/GI: Normal
- Genitourinary: Deferred
- **Rectal:** Deferred
- **Extremities:** Non-localized tenderness without edema to the bilateral lower extremities, tenderness diffusely to the upper extremities, no signs of trauma
- **Back:** Bilateral lumbar paraspinal muscle tenderness, no midline tenderness
- **Neuro:** Normal
- **Skin:** Normal
- **Lymph:** Normal
- **Psych:** Agitated, otherwise normal

### Critical Actions and Cueing Guidelines

1. **Critical Action 1:** *Patient requires oxygen administration and should concurrently be placed in a resuscitation room*.
  - Cueing Guideline (if applicable): RN may state, “His pulse-ox keeps dropping; do you want me to call respiratory?”
2. **Critical Action 2:** *Patient requires IV access with appropriate analgesic administration. This includes an IV opiate medication and is not satisfied with oral medications or IV NSAIDs*.
  - Cueing Guideline (if applicable): Patient will become increasingly uncooperative with the history and/or physical exam until analgesia is provided.
3. **Critical Action 3:** *Patient should be started on broad spectrum antibiotics after his chest radiograph is viewed, and blood cultures should be obtained*.
  - Cueing Guideline (if applicable): RN may state, “Dr., do you want any additional labs before starting the antibiotics?” If antibiotics are not initiated, the patient should develop a fever of 38.2 °C.
4. **Critical Action 4:** *Consult placed to hematology*.
  - Cueing Guideline (if applicable): The admitting team may state, “Is anyone else involved in his care?”
5. **Critical Action 5:** *Admit to the medical intensive care unit*.
  - Cueing Guideline (if applicable): If the learner attempts to admit to the hospitalist, primary care doctor, hematologist, or other non-intensivist, the clinician should ask if they think the patient is stable enough for a floor bed.

## ORAL BOARDS ASSESSMENT

*Acute Chest Syndrome*

Learner: _________________________________________

**Critical Actions:**

□ Patient requires oxygen administration and should concurrently be placed in a resuscitation room
□ Patient requires IV access with appropriate analgesic administration. This includes an IV opiate medication and is not satisfied with oral medications or IV NSAIDs.
□ Patient should be started on broad spectrum antibiotics after his chest radiograph is viewed, and blood cultures should be obtained.
□ Consult placed to hematology.
□ Admit to the medical intensive care unit.

**Summative and formative comments:**

**Milestone assessment:**

### Stimulus Inventory

**Stimulus #1**

### Patient Information

**Vital Signs:**

**Stimulus #2**

### Arterial Blood Gas

**Stimulus #3**

### Complete Blood Count (CBC)

**Differential:**

**Stimulus #4**

### Basic Metabolic Panel (BMP)

**Stimulus #5**

### Cardiac Labs

**Stimulus #6**

### Coagulation panel

**Stimulus #7**

### Chest Radiograph (CXR)

**Stimulus #8**

[Fig f1-jetem-8-1-o1] [Fig f2-jetem-8-1-o1]

## DEBRIEFING AND EVALUATION PEARLS

### Acute Chest Syndrome

1. Consider acute chest syndrome in patients with sickle cell disease who present to the emergency department with fever and cardiorespiratory symptoms.^6^
2. Pathophysiology of acute chest syndrome involves vaso-occlusion in the pulmonary vasculature, release of pro-inflammatory mediators, and infarction of lung tissue.^1^,^2^,^5^, ^6^
3. Common precipitants of acute chest syndrome include infection, pain crises, fat embolism, rib infarction, and atelectasis from hypoventilation.^1^,^2^
4. Acute chest syndrome occurs most commonly in children ages two to four years of age, and has an 80% recurrence rate.^5^
5. Diagnosis of acute chest syndrome is made by chest radiograph or chest CT with a new infiltrate and any of the following: fever, dyspnea, cough, wheezing, hypoxia, or chest pain.^5^, ^6^
6. Management is multifactorial:
  - Supplemental oxygen for hypoxia, targeting SpO2 > 92%
  - Hydration with hypotonic IV fluids
  - IV Antibiotics covering *Mycoplasma* and *Chlamydia Pneumoniae*
  - Analgesia to prevent splinting and atelectasis in addition to aggressive pulmonary toilet
  - Consideration of transfusion 6, 8
7. Transfusion in acute chest syndrome is guided by empiric evidence and should be undertaken in consultation with a hematologist. Patients with low hemoglobin may benefit from simple transfusion, whereas patients with acute chest syndrome and elevated hemoglobin may require exchange transfusion.^9^
